# Integrin Mac1 mediates paraquat and maneb-induced learning and memory impairments in mice through NADPH oxidase–NLRP3 inflammasome axis-dependent microglial activation

**DOI:** 10.1186/s12974-023-02732-x

**Published:** 2023-02-18

**Authors:** Liyan Hou, Jianing Liu, Fuqiang Sun, Ruixue Huang, Rui Chang, Zhengzheng Ruan, Ying Wang, Jie Zhao, Qingshan Wang

**Affiliations:** 1 Dalian Medical University Library, Dalian Medical University, No. 9 W. Lvshun South Road, Dalian, 116044 China; 2 National-Local Joint Engineering Research Center for Drug-Research and Development (R & D) of Neurodegenerative Diseases, Dalian Medical University, Dalian, 116044 China; 3 School of Public Health, Dalian Medical University, No. 9 W. Lvshun South Road, Dalian, 116044 China

**Keywords:** Integrin, Pesticide, Learning and memory deficits, NLRP3 inflammasome, NADPH oxidase, Microglial activation, Parkinson’s disease

## Abstract

**Introduction:**

The mechanisms of cognitive impairments in Parkinson’s disease (PD) remain unknown. Accumulating evidence revealed that brain neuroinflammatory response mediated by microglial cells contributes to cognitive deficits in neuropathological conditions and macrophage antigen complex-1 (Mac1) is a key factor in controlling microglial activation.

**Objectives:**

To explore whether Mac1-mediated microglial activation participates in cognitive dysfunction in PD using paraquat and maneb-generated mouse PD model.

**Methods:**

Cognitive performance was measured in wild type and Mac1^−/−^ mice using Morris water maze test. The role and mechanisms of NADPH oxidase (NOX)–NLRP3 inflammasome axis in Mac1-mediated microglial dysfunction, neuronal damage, synaptic degeneration and phosphorylation (Ser129) of α-synuclein were explored by immunohistochemistry, Western blot and RT-PCR.

**Results:**

Genetic deletion of Mac1 significantly ameliorated learning and memory impairments, neuronal damage, synaptic loss and α-synuclein phosphorylation (Ser129) caused by paraquat and maneb in mice. Subsequently, blocking Mac1 activation was found to mitigate paraquat and maneb-elicited microglial NLRP3 inflammasome activation in both in vivo and in vitro. Interestingly, stimulating activation of NOX by phorbol myristate acetate abolished the inhibitory effects of Mac1 blocking peptide RGD on paraquat and maneb-provoked NLRP3 inflammasome activation, indicating a key role of NOX in Mac1-mediated NLRP3 inflammasome activation. Furthermore, NOX1 and NOX2, two members of NOX family, and downstream PAK1 and MAPK pathways were recognized to be essential for NOX to regulate NLRP3 inflammasome activation. Finally, a NLRP3 inflammasome inhibitor glybenclamide abrogated microglial M1 activation, neurodegeneration and phosphorylation (Ser129) of α-synuclein elicited by paraquat and maneb, which were accompanied by improved cognitive capacity in mice.

**Conclusions:**

Mac1 was involved in cognitive dysfunction in a mouse PD model through NOX–NLRP3 inflammasome axis-dependent microglial activation, providing a novel mechanistic basis of cognitive decline in PD.

**Supplementary Information:**

The online version contains supplementary material available at 10.1186/s12974-023-02732-x.

## Introduction

Chronically exposed to paraquat and maneb leads to elevated incidence of Parkinson’s disease (PD) in human [1]. Rodents exposed to paraquat and maneb also recapitalize the main features of PD, such as nigral dopaminergic neurodegeneration and motor dysfunction, which are gradually accepted as animal models of PD [2]. Recent evidence revealed that the pathological alterations of PD are not limited in nigrostriatal system and neuronal damage in cortex and hippocampus **is** also detected [3, 4]. PD patients also experience both motor and non-motor dysfunctions [5]. Cognitive deficits including learning and memory dysfunction are common non-motor dysfunction in PD and occur in more than 80% of patients in the late stage of PD progression [6]. In clinic, learning and memory impairments are usually inadequately treated and gradually become a key determinator of life quality of PD patients [7, 8]. We recently reported that paraquat and maneb coexposure could induce learning and memory dysfunction in mice [9]; however, the mechanisms remain unclear.

Neuroinflammation has long been recognized to be essential in driving PD progression [10]. Glia cells, especially microglia, are key players in mediating brain neuroinflammatory response in neurological disorders [11]. Activated microglia and the production of proinflammatory cytokines are detected not only in nigrostriatal regions of PD patients but also in brain areas that contribute to cognition, such as hippocampus and cortex [12]. Femminella and colleagues reported that microglial activation negatively correlated with volume of hippocampus and cerebral glucose metabolic rate in patients with PD dementia [13]. Furthermore, compared with control subjects, PD patients also exhibit high levels of tumor necrosis factor α (TNFα) in both plasma and cerebrospinal fluid [14, 15], which is accompanied with their poor cognitive performance [16, 17]. Similarly, significant correlation between cognitive dysfunction and anterior temporal microglial activation was also observed in Alzheimer's disease patients [18].

Macrophage antigen complex-1 (Mac1; CR3; CD11b/CD18), a member of β2 integrin family, displays high expression in microglia [19]. Strong evidence reveals a key role of Mac1 in regulating microglial activation in PD. The expression levels of Mac1 are elevated in brains of PD patients and multiple rodent models [20, 21]. Furthermore, genetic deletion or pharmacological inactivation of Mac1 abrogates microglial activation provoked by α-synuclein, an important component of Lewy body in PD [22]. Genetic ablation of Mac1 also abrogates microglial activation, proinflammatory factors production, dopaminergic neuron loss and behavior impairments in MPTP-lesioned mice [23]. A recent report revealed that paraquat and maneb intoxication results in microglial activation in primary midbrain cultures, which is markedly mitigated by a Mac1 neutralizing antibody [24]. However, whether Mac1 is involved in paraquat and maneb-elicited learning and memory impairments by regulating microglia-mediated neuroinflammation remains to be investigated.

To this end, we compared the learning and memory performance, neuronal damage and phosphorylation (Ser129) of α-synuclein between wild type (WT) and Mac1^−/−^ mice injected with paraquat and maneb (referred to subsequently as P + M). Then, we investigated the underlying mechanisms of Mac1-mediated microglial activation in paraquat and maneb-elicited learning and memory impairments. Our results suggested that Mac1 through NADPH oxidase (NOX)–NLRP3 inflammasome axis regulates microglial activation and M1 polarization to mediate cognitive impairments in PD.

## Materials and methods

### Reagents

Paraquat, glybenclamide, maneb, phorbol myristate acetate (PMA) 3,3′-diaminobenzidine (DAB) were provided by Sigma-Aldrich, Inc (St. Louis, MO, USA). The AG RNAex Pro Reagent, Pro Taq HS qPCR Kit and SYBR Green Premix were provided by Accurate Biotechnology (Hunan, China). The RGD peptide was provided by Selleck (Shanghai, China). The antibodies against Neu-N, postsynaptic density protein 95 (PSD-95) and tyrosine hydroxylase (TH) were provided by EMD Millipore (Temecula, CA, USA). The antibodies against NLRP3, Mac1, total and phosphorylated (Ser129) α-synuclein were provided by Abcam (Cambridge, MA, USA). Cell Signaling Technology (MA, USA) provides antibodies against caspase-1, and interleukin-1β (IL-1β), p–p38, p38, p-JNK, JNK, p-ERK, ERK, p-P21-activated kinase 1 (PAK1) and PAK1. GenePharma (Shanghai, China) provides NOX1 and NOX2-specific siRNAs. Biological Industries (Cromwell, CT, USA) provides the ECL reagents.

### Animal dosing

Eight-week-old male C57BL/6 and Mac1^−/−^ mice were randomly assigned into vehicle (control) and P + M-injected groups. Paraquat and maneb dissolved in sterile PBS were administered (i.p.) to mice for 6 weeks (twice/week). The administration dosage of paraquat and maneb was 10 mg/kg and 30 mg/kg, respectively [25]. Vehicle mice received an equal amount of PBS. The test of Morris water maze (MWM) was conducted after last P + M injection. And then, mice were sacrificed and the brains were dissected.

### Ethics statement

All experiments involving animals were conducted according to the ethical policies and their care were approved by the Institutional Animal Care and Use Committee of Dalian Medical University, China.

### Gybenclamide administration

Glybenclamide dissolved in DMSO (0.1%) was administered to mice (i.p., 1 mg/kg) 30 min before P + M injection for 6 weeks (two times per week) [26, 27].

### MWM test

MWM includes navigation and spatial probe tests [9]. Briefly, in navigation test, a transparent cylindrical three-dimensional platform was placed 1 cm under the water surface in the target quadrant. Mice in each group were permitted to find the platform within 90 s once they were put in one of four quadrants. If they failed, mice were guided to find the platform and their escape latency was considered as 90 s. Four trials were performed in each mouse with a 5 min break every day and sustained for 4 consecutive days. After that, the spatial probe test was conducted by removing the platform from the maze. Mice freely swam in the maze for 1 min. Parameters for learning and memory capacities were analyzed by an intelligent video tracking system (NoldusEtho Vision system).

### Immunohistochemistry

For immunohistochemistry analyses, the brains were dissected from mice after perfusion with 4% paraformaldehyde (PFA). Samples were further post-fixed with 4% PFA for 48 h, followed by 30% sucrose solution for additional 48 h. Thirty micrometer floating sections covering both hippocampus and cortex regions were prepared for immunostaining [21, 28]. Briefly, brain sections were initially incubated with 3% H_2_O_2_ to inactive peroxidase, followed by 4% goat serum diluted by 0.25% Triton/PBS. The primary antibodies against Mac1, Iba-1, Neu-N and PSD-95 were used for incubation with the sections for 24 h (4 °C). Sections were probed with appropriate secondary antibodies for 2 h after washing three times. The binding signals were detected by a Vectastain ABC kit and subsequent DAB incubation based on the protocol provided by manufacturer.

Two individuals not involved in this experiment performed quantitative analyses of Neu-N^+^ cell number in sections using automated counting function in ImageJ [29, 30]. The quantitative analyses of PSD-95, Iba-1 and Mac1 immunostaining were conducted from three to four sections, spaced 120 μm apart, in each mouse using ImageJ [31, 32].

### BV2 microglia

The BV2 microglia were routinely maintained in DMEM medium with 10% FBS and 1% penicillin/streptomycin at 37 °C supplemented with 5% CO_2_ [24].

### siRNA transfection

Mouse NOX1 or NOX2-specific siRNAs were transfected into BV2 cells using RNAi-Mate transfection reagent in Opti-MEM reduced serum media. Forty-eight hours later, the efficacy of siRNAs were tested by examining the expression levels of NOX1 and NOX2 as described previously [33].

### BV2 treatment

For Western blot analyses, cells were treated with RGD, anti-Mac1 blocking antibody or NOX inhibitor apocynin for 30 min before P + M. After 24 h of P + M intoxication, cells were collected. For signaling pathway analyses, apocynin was administered to cells for 30 min, followed **by** P + M intoxication for additional 1 h. Cells among groups were lysed in RIPA buffer and then were homogenized (20–25 stokes, tight pestle A). The supernatant was collected after 15 min of centrifugation (10,000×*g*) and was used for subsequent analyses.

### Western blot

Mice were euthanized and were transcardially perfused with PBS only. Hippocampal and cortical samples were dissected quickly and were homogenized in ice-cold RIPA buffer, followed by centrifugation for 15 min at 10,000×*g*. The protein concentrations were measured by BCA protein quantification kit and then the same amount of protein for each group was subjected to 4–12% SDS–PAGE as described previously [24, 34]. The immunoblot analyses were conducted using primary antibodies against Neu-N, PSD-95, TH, IL-1β, NLRP3, caspase-1, p-p38, p38, p-JNK, JNK, p-ERK, ERK, p-PAK1, PAK1, α-synuclein and p (Ser129) α-synuclein at 4 °C for 24 h and HRP-linked secondary antibody (1:3000) for additional 2 h at RT. The signals were captured by ECL reagents and the quantitative analyses of blot density were conducted using ImageJ.

### Real-time PCR

Hippocampal and cortical samples were dissected from mice transcardially perfused with PBS only. RNA in each brain samples was purified using AG RNAex Pro Reagent based on manufacture’s protocol. One microgram RNA was used for cDNA synthesis [22, 25]. RT-PCR amplification was conducted using SYBR Green Premix Ex TaqTMII and Takara Thermal Cycler Dice™ Real Time System. The PCR conditions were 95 ºC for 10 s, 55 ºC for 30 s, and 72 ºC for 30 s for total 40 cycles. The alteration of mRNA level of genes was normalized to GAPDH.

### Statistical analysis

Results were presented as mean ± SEM. The Shapiro–Wilk and Bartlett’s tests were used to differentiate normal distribution and equal variance of data, respectively. For normally distributed data, one-way or two-way ANOVA, followed by Tukey’s post hoc test were employed for comparisons; otherwise, a nonparametric test was employed. Data generated from MWM test were analyzed using repeated measures ANOVA. *P* < 0.05 was defined as significant.

## Results

### P + M exposure elevates Mac1 expression

**Fig. 1:**
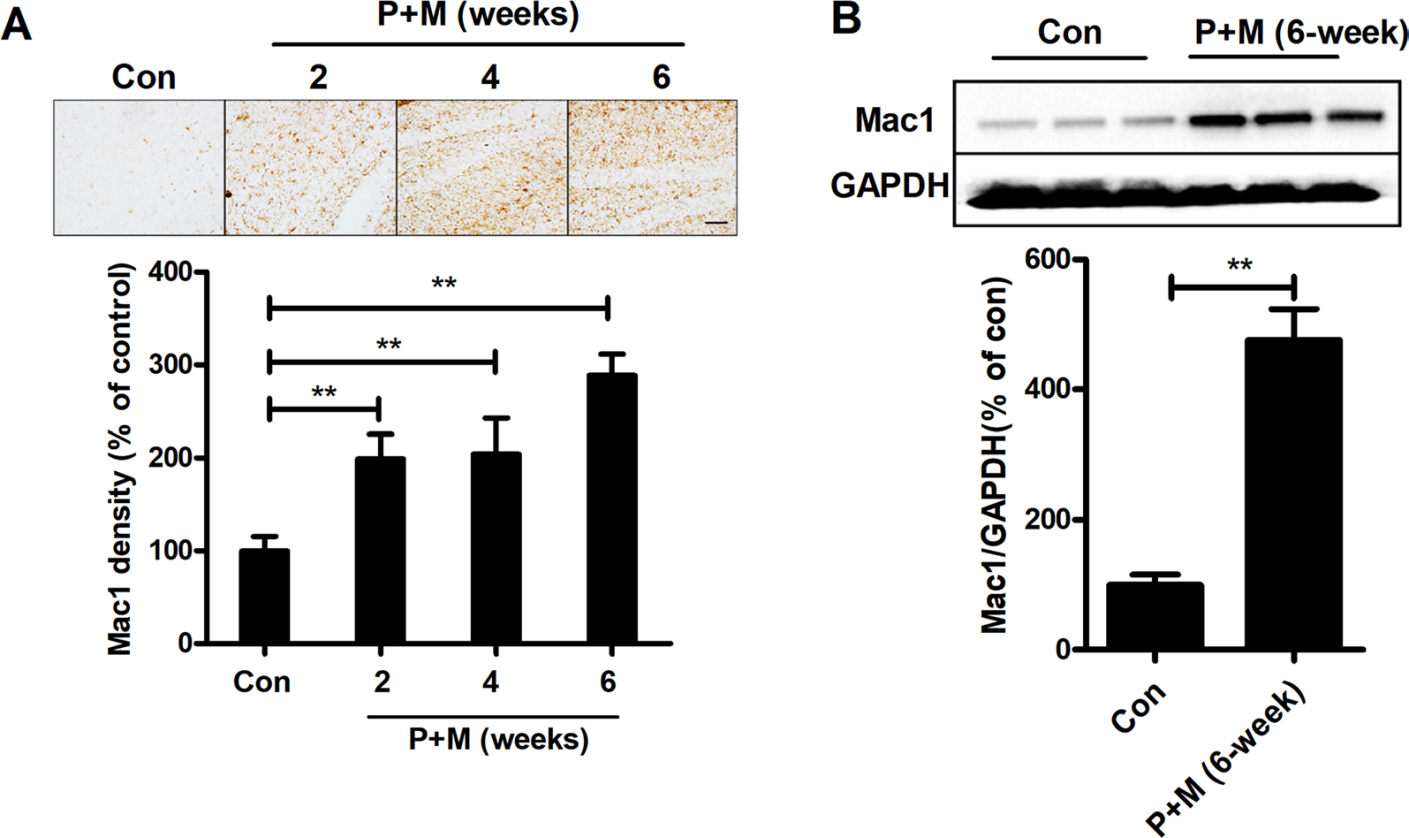
P + M exposure upregulates Mac1 expression in the hippocampus of mice. **A** Representative pictures of Mac1 staining in the hippocampus of mice and quantification of Mac1 immunostaining. **B** Representative blots of Mac1 and the quantification of the blots. *n* = 3; **p < 0.01; Scale bar = 100 μm

To determine whether Mac1 contributes to learning and memory deficits in PD, P + M were used to generate a mouse model. Significant loss of nigral dopaminergic neuron was observed in P + M-injected mice [24, 35], supporting a PD mouse model was established in our condition. The expression of Mac1 was initially determined in mice. Anti-Mac1 immunohistochemistry revealed elevated expression level of Mac1 in the hippocampus of P + M-intoxicated mice in comparison with controls (Fig. 1A). Quantification of Mac1 immunostaining density confirmed the observation (Fig. 1A). The alterations of Mac1 were further determined by Western blot. Consistently, we found that in comparison with controls, P + M-injected mice displayed increased expression levels of Mac1 in the hippocampus (Fig. 1B). Since cortex is also considered to contribute to cognitive impairment in PD patient [36], the expression of Mac1 was also determined in the cortex brain region. In agreement with that of hippocampus, the increased expression of Mac1 was also detected in the cortex of mice intoxicated with P + M compared with vehicle (Additional file 1: Fig S1).

### Genetic deletion of Mac1 alleviates P + M-elicited learning and memory impairments

**Fig. 2:**
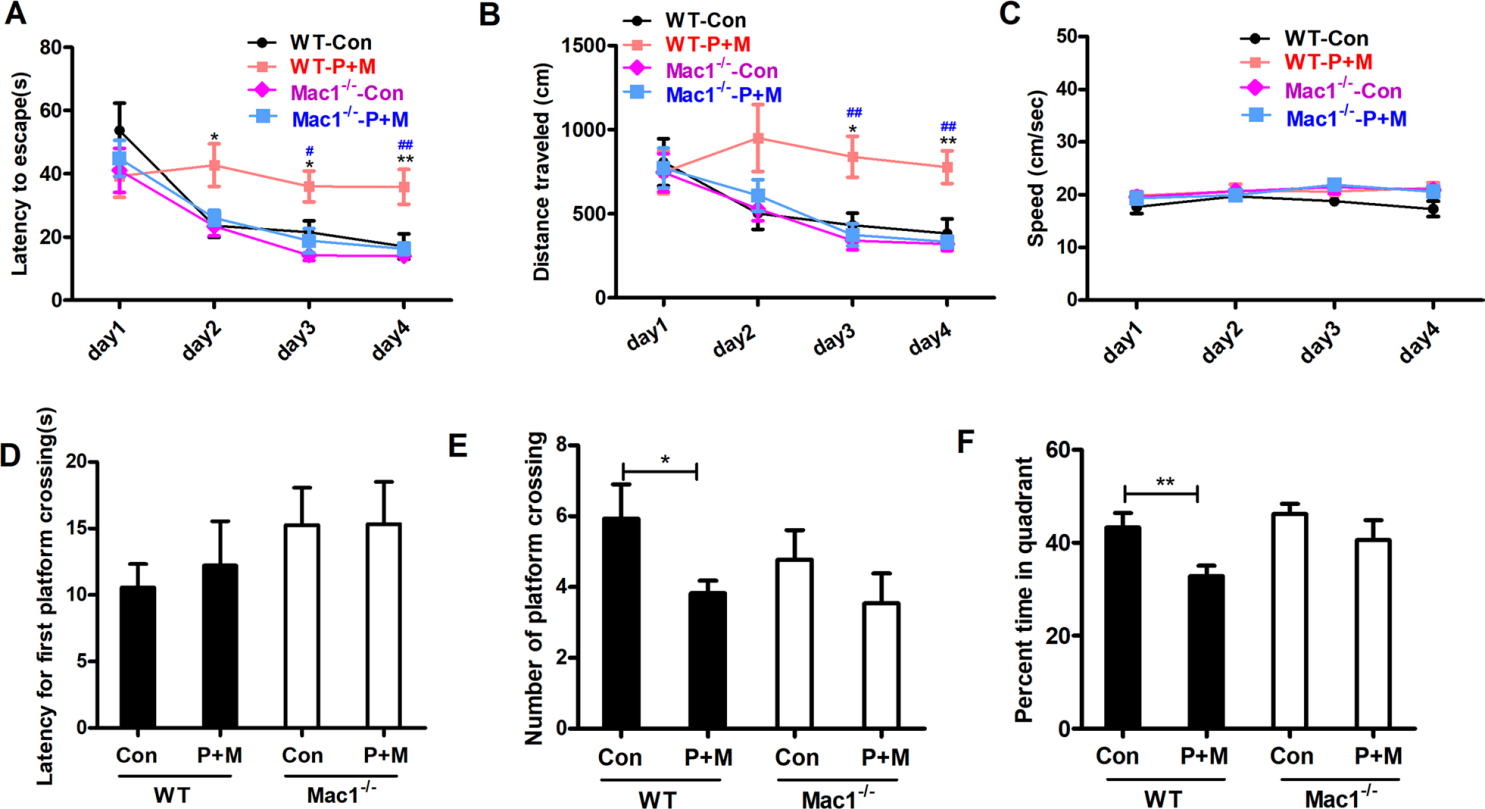
Genetic deletion of Mac1 ameliorates cognitive impairments in P + M-lesioned mice. **A** Escape latency, **B** traveled distance and (**C**) swimming speed of WT and Mac1^−/−^ mice. **D** First platform crossing latency, (**E**) platform crossing number and (**F**) time percentage in target quadrant of WT and Mac1^−/−^ mice. *n* = 13–16; *p < 0.05, **p < 0.01 for comparison between Con & P + M groups; ^#^*p* < 0.05, ^##^*p* < 0.01 for comparison between WT-P + M & Mac1^−/−^-P + M groups

We then compared the learning and memory capacity between WT and Mac1^−/−^ mice after P + M treatment. As illustrated in Fig. 2A, B, control mice in both strains displayed gradual decrease of escape latency in four consecutive days of acquisition trials. Consistently, the traveled distance of control mice also decreased gradually, indicating normal spatial learning capacity. P + M exposure impaired spatial learning ability in WT mice by showing extended escape latency and enlarged traveled distance in comparison with controls. Interestingly, P + M-elicited learning dysfunction were significantly attenuated in Mac1^−/−^ mice compared with WT mice (Fig. 2A, B). Similar escape latency and traveled distance were observed in P + M and vehicle-treated Mac1^−/−^ mice (Fig. 2A, B). No difference in swimming speed of mice excluded the possibility that the differences in acquisition trials between WT and Mac1^−/−^ mice were caused by locomotor deficits (Fig. 2C).

The effects of Mac1 on spatial memory ability were further investigated. In comparison with vehicle, P + M treatment greatly reduced platform crossing number and the percent time in target quadrant in WT mice, although the first platform crossing latency reminded unchanged (Fig. 2D–F). Consistently, P + M-induced decrease of platform crossing number and percent time in target quadrant was significantly mitigated in Mac1^−/−^ mice (Fig. 2E, F).

### Genetic deletion of Mac1 attenuates neurodegeneration, synaptic damage and phosphorylation (Ser129) of α-synuclein elicited by P + M

**Fig. 3:**
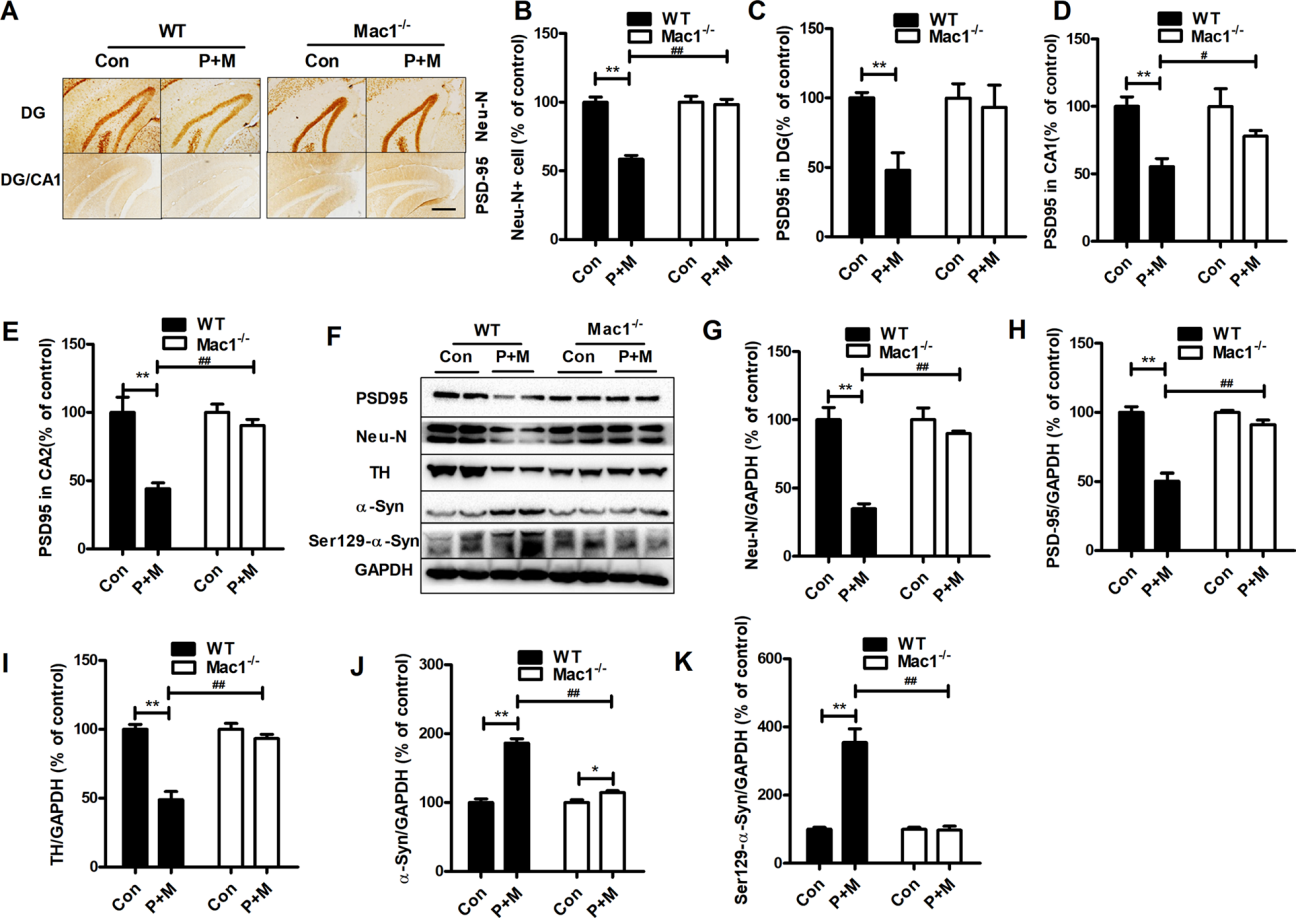
Genetic deletion of Mac1 mitigates P + M-elicited neuronal damage, synaptic loss and phosphorylation (Ser129) of α-synuclein in mice. **A** Representative pictures of PSD-95 and Neu-N staining in WT and Mac1^−/−^ mice. **B** Quantification of Neu-N^+^ cells. **C**–**E** Quantification of optical density of PSD-95 staining. **F** Representative blots of PSD-95, Neu-N, TH, toal and phosphorylated (Ser129) α-synuclein in mice. **G–K** Quantification of the blots. *n* = 4–6; **p < 0.01 for comparison between Con & P + M groups; ^#^*p* < 0.05, ^##^*p* < 0.01 for comparison between WT-P + M & Mac1^−/−^-P + M groups; Scale bar = 200 μm

Clinical studies demonstrated that loss of neurons and synapses as well as α-synuclein aggregation and phosphorylation are essential for PD dementia [36–38]. The effects of Mac1 on neuron damage were, therefore, investigated. In comparison with control, P + M injection resulted in decrease of Neu-N^+^ cell number in the hippocampal region in WT mice (Fig. 3A, B). In contrast, the loss of hippocampal Neu-N^+^ neurons induced by P + M was greatly attenuated in Mac1^−/−^ mice compared with WT mice (Fig. 3A, B), indicating reduced neurodegeneration. This conclusion was further supported by Western blot analyses, in which a high expression level of Neu-N was detected in P + M-injected Mac1^−/−^ mice compared with WT mice treated with P + M (Fig. 3F, G).

The loss of synapse and interaction between dopaminergic system and hippocampus play important roles in cognitive decline in some PD patients [39–41]. We thus investigated the effects of Mac1 on expression of PSD-95, a post-synaptic marker, and TH, a marker for dopaminergic neuron [42], in mice after P + M exposure. Immunostaining analysis showed a marked reduction of PSD-95 expression in the hippocampus (DG, CA1 and CA2 regions) in WT mice injected with P + M (Fig. 3A, C, D, E). However, the reduced PSD-95 expression in P + M-injected Mac1^−/−^ mice was not noted (Fig. 3A, C, D, E). Analyses of PSD-95 expression by Western blot further supported these observations (Fig. 3F, H). Previous study indicated that cortical neurodegeneration may also contribute to cognitive deficits in PD [43]. In agreement with reduced neurodegeneration and synaptic loss in the hippocampus, Mac1^−/−^ mice were more resistant to P + M-elicited neuronal loss and reduction of PSD-95 expression in cortex than WT mice (Additional file 1: Fig. S2). Consistently, the reduced expression of TH induced by P + M was also attenuated in Mac1^−/−^ mice compared with WT group (Fig. 3F, I).

The effects of Mac1 on α-synuclein pathology elicited by P + M were further investigated. We compared α-synuclein expression level between in P + M-injected WT and Mac1^−/−^ mice. Figure 3F, J shows that P + M injection led to increase of α-synuclein expression in WT mice, which was markedly attenuated in Mac1^−/−^ mice. The phosphorylation of Ser129 is important for neurotoxicity of α-synuclein [44]. Consistently, P + M-induced expression of Ser129-phosphorylated α-synuclein was also mitigated in Mac1^−/−^ mice compared with WT group (Fig. 3F, K).

### Genetic deletion of Mac1 abrogates microglial activation elicited by P + M

**Fig. 4:**
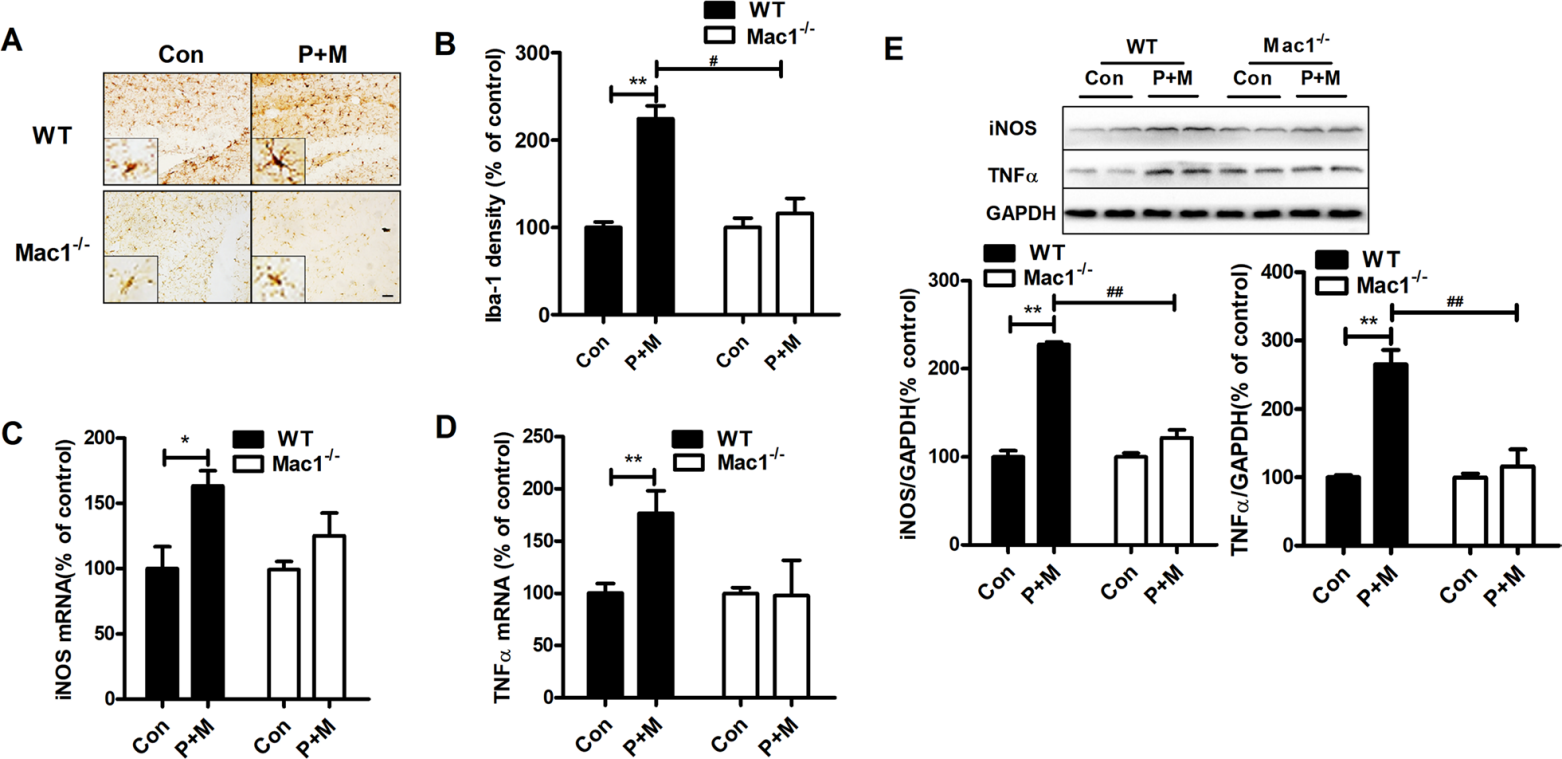
Genetic deletion of Mac1 impedes P + M-elicited neuroinflammation. **A** Representative pictures of Iba-1 staining in WT and Mac1^−/−^ mice. **B** Quantification of Iba-1 immunostaining. (**C**, **D**) The mRNA levels of iNOS (**C**) and TNFα (**D**) in WT and Mac1^−/−^ mice. **E** Representative blots of iNOS and TNFα in mice and the quantification of the blots. *p < 0.05. **p < 0.01 for comparison between Con & P + M groups; ^#^*p* < 0.05 for comparison between WT-P + M & Mac1^−/−^-P + M groups; Scale bar = 50 μm

To explore whether microglia-mediated neuroinflammation contributes to Mac1-mediated cognitive deficits in P + M-injected mice, microglial activation was determined. Immunohistochemistry revealed Iba-1-identified and hypertrophic microglia in WT mice injected with P + M, indicating microglial activation. However, microglia in P + M-intoxicated Mac1^−/−^ mice displayed ramified morphology and decreased immunostaining of Iba-1 (Fig. 4A), suggesting that Mac1 deficiency attenuates activation of microglia. Optical density analysis of Iba-1 immunostaining supported this conclusion (Fig. 4B).

Activated microglia induce neuronal damage through the release of a variety of proinflammatory factors. We further determined the gene expression of TNFα and iNOS. We found that P + M led to significant upregulation of TNFα and iNOS mRNA levels in WT mice, which were greatly suppressed in Mac1^−/−^ mice (Fig. 4C, D). These findings suggest that Mac1 deficiency mitigates P + M-induced neuroinflammation.

### Genetic deletion of Mac1 mitigates NLRP3 inflammasome activation elicited by P + M

**Fig. 5:**
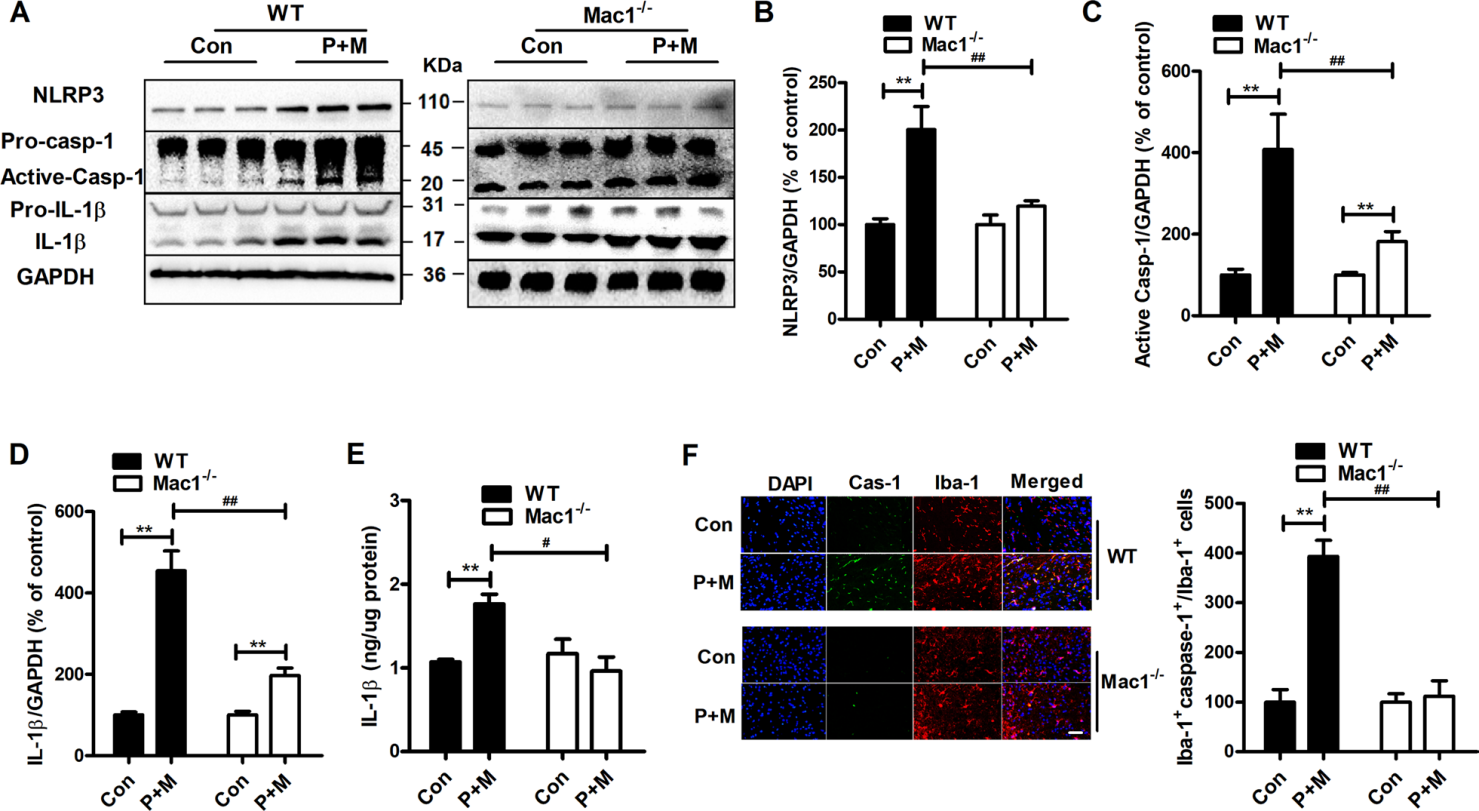
Genetic deletion of Mac1 mitigates P + M-elicited NLRP3 inflammasome activation in mice. **A** Representative blots of NLRP3, active caspase-1 and mature IL-1β in WT and Mac1^−/−^ mice. *n* = 6 **B**–**D** Quantification of the blots. **E** Contents of IL-1β in mice. **F** Representative images of double caspase-1 and Iba-1 fluorescence staining in P + M-injected WT and Mac1^−/−^ mice. *n* = 3. **p < 0.01 for comparison between Con & P + M groups; ^#^*p* < 0.05, ^##^*p* < 0.01 for comparison between WT-P + M & Mac1^−/−^-P + M groups; Scale bar = 50 μm

Next, we determined the mechanisms of how Mac1 regulates microglial activation. Previous report revealed a pivotal role of NLRP3 inflammasome in controlling microglial activation and related cognitive performance in a mouse AD model [45]. We then investigated whether NLRP3 inflammasome contributes to Mac1-mediated activation of microglia. In comparison with control, P + M intoxication resulted in elevation of expression of NLRP3 in WT mice (Fig. 5A, B). Elevated expression of active caspase-1, mature IL-1β and IL-1β contents were also noted in P + M-injected WT mice (Fig. 5A, C–E), indicating NLRP3 inflammasome activation. However, the elevation of NLRP3, active caspase-1 expression and mature IL-1β levels elicited by P + M were significantly reduced in Mac1^−/−^ mice compared with WT mice (Fig. 5A–E). Furthermore, no significant difference of IL-1β content in Mac1^−/−^ mice after P + M and saline injection was observed (Fig. 5E).

To investigate whether attenuated NLRP3 inflammasome activation in Mac1^−/−^ mice occurred in microglia, antibodies against caspase-1 and Iba-1 were used to conduct double immunofluorescence staining. Consistent with that of Western blot, P + M exposure induced expression of caspase-1 in WT mice. Co-staining with Iba-1 revealed a colocalization of caspase-1 and Iba-1 (Fig. 5F), indicating increased expression of microglial caspase-1. However, microglia in Mac1^−/−^ mice failed to express caspase-1 after P + M treatment (Fig. 5F).

### NOX contributes to Mac1-mediated NLRP3 inflammasome activation

**Fig. 6:**
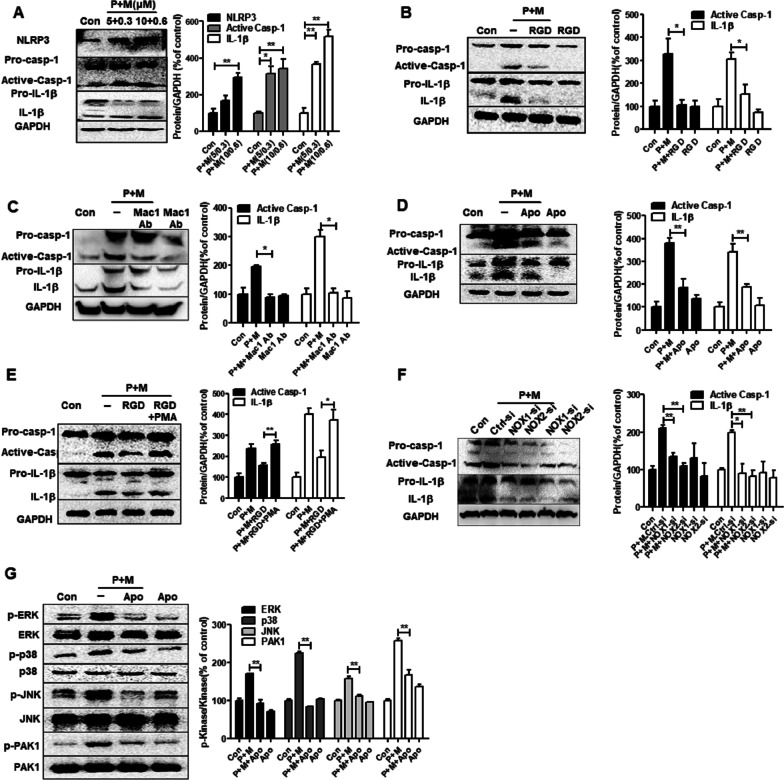
NOX contributes to Mac1-mediated NLRP3 inflammasome activation in microglia intoxicated with P + M. **A** Representative blots of NLRP3, active caspase-1 and mature IL-1β in BV2 microglia treated with P + M for 24 h and the quantification of the blots. The concentrations of P + M were 5 + 0.3 and 10 + 0.6 μM, which were chosen based on previous report [24]. **B** Representative blots of active caspase-1 and mature IL-1β in P + M-treated BV2 microglia with or without RGD (10 μM) pretreatment (30 min) and the quantification of density of these blots. **C** Representative blots of active caspase-1 and mature IL-1β in P + M-treated BV2 microglia with or without anti-Mac1 Ab (1 μg/ml) pretreatment (30 min) and the quantification of density of these blots. **D** Representative blots of active caspase-1 and mature IL-1β in P + M-treated BV2 microglia with or without apocynin (0.25 mM) pretreatment (30 min) and the quantification of density of these blots. **E** Representative blots of active caspase-1 and mature IL-1β in P + M-treated BV2 microglia with or without RGD and PMA (100 ng/ml) pretreatment (30 min) and the quantification of density of these blots. **F** Representative blots of active caspase-1 and mature IL-1β in NOX1 and NOX2 silenced BV2 microglia treated with P + M and the quantification of density of these blots. **G** Representative blots of phosphorylated and nonphosphorylated ERK1/2, p38, JNK and PAK1 in P + M-treated BV2 microglia with or without apocynin pretreatment (30 min) and the quantification of density of these blots. *n* = 3; *p < 0.05, **p < 0.01

We recently reported that Mac1 is essential for NOX activation and related superoxide release in microglia intoxicated with P + M [24]. To investigate whether NOX contributes to Mac1-mediated activation of NLRP3 inflammasome, BV2 microglia were employed. In agreement with that of in vivo, P + M dose-dependently elicited NLRP3 inflammasome activation (Fig. 6A), which was markedly mitigated by Mac1 blocking peptide RGD and an inhibitory antibody (Fig. 6B, C). Interestingly, NOX inhibitor apocynin also blocked NLRP3 inflammasome activation in P + M-intoxicated cultures (Fig. 6D). More importantly, activating NOX by PMA, a classic activator of NOX through PKC [46], restored NLRP3 inflammasome activation in RGD and P + M co-treated microglial cells (Fig. 6E), indicating a key role of NOX in Mac1-mediated NLRP3 inflammasome activation.

To differentiate the contribution of NOX sub-members in Mac1-regulated activation of NLRP3 inflammasome, specific siRNAs were used to silence NOX1 and NOX2, two main members of NOX family expressed in microglia [47]. As shown in Fig. 6F, NOX1 and NOX2 knockdown also abrogated P + M-elicited NLRP3 inflammasome activation since reduced expressions of NLRP3, active caspase-1 and IL-1β were observed in BV2 microglia treated with combined NOX1-/NOX2-siRNAs and P + M in comparison with ctrl-siRNA and P + M group.

MAPK and PAK1 pathways have been implicated in controlling NLRP3 inflammasome activation induced by either exogenous or endogenous ligands [48]. In comparison with vehicle, P + M intoxication resulted in an elevated expression of phosphorylated ERK, JNK, p38 and PAK1 in microglial cells (Fig. 6G). NOX inhibitor apocynin significantly impeded P + M-elicited phosphorylation of ERK, JNK, p38 and PAK1 (Fig. 6G), indicating attenuated activation of MAPK and PAK1 pathways.

To determine whether the key results drawn from BV2 cells could be replicated in primary microglia, the effects of Mac1 blocking antibody, apocynin and combined Mac1 blocking antibody and PMA on P + M-induced NLRP3 activation were investigated in primary cultures (Additional file 1: Fig. S3). Consistent with that of BV2 microglia, P + M-induced expression of NLRP3, active caspase-1 and IL-1β was significantly blocked by Mac1 blocking Ab and apocynin Additional file 1: Fig. S3A, B). PMA reversed the inhibition of Mac1 blocking antibody against P + M-induced activation of NLRP3 inflammasome (Additional file 1: Fig. S3C). Furthermore, the inhibition of apocynin on P + M-induced ERK phosphorylation was also observed (Additional file 1: Fig. S3D).

### Glybenclamide counteracts microglial M1 activation elicited by P + M

**Fig. 7:**
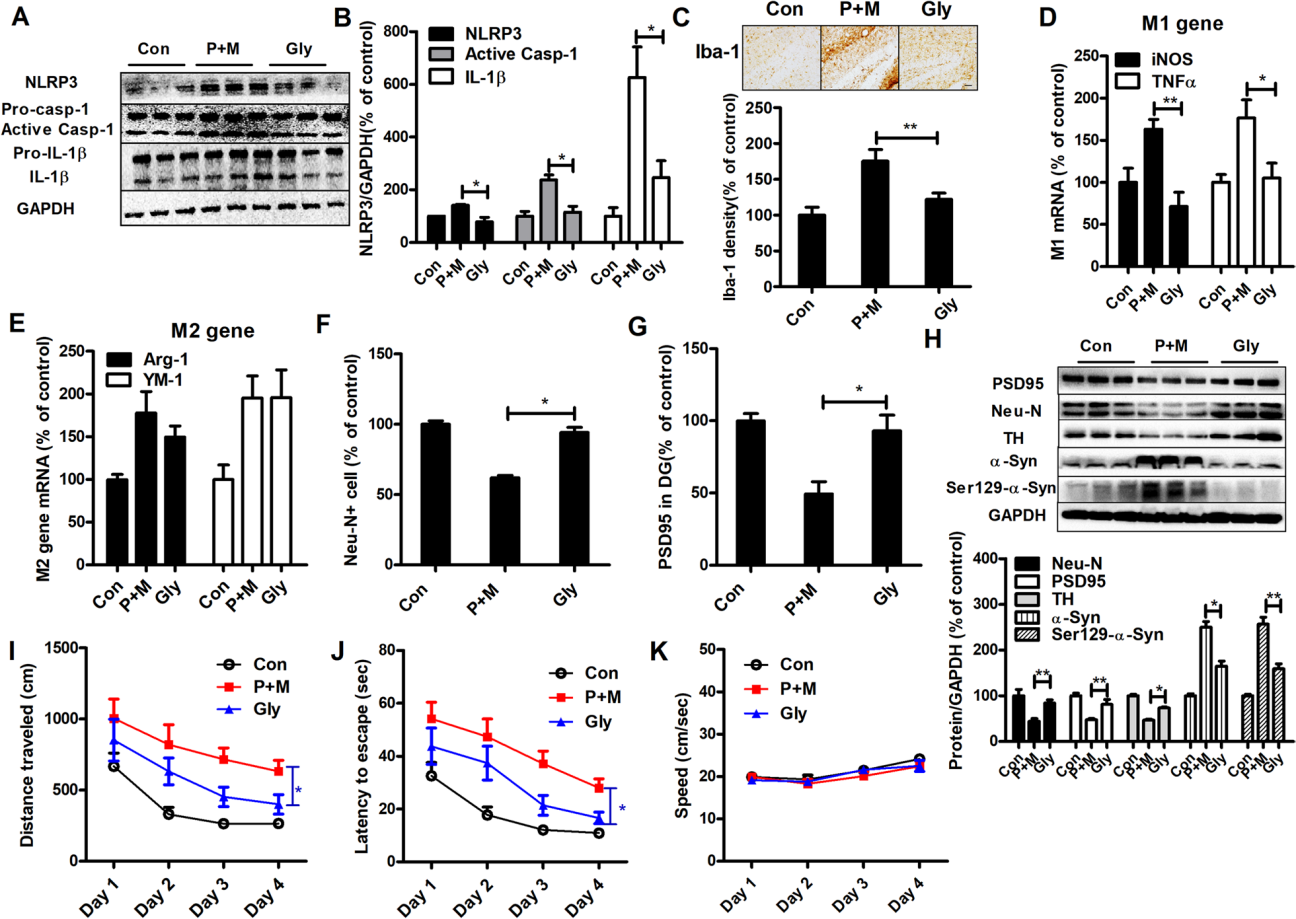
Glybenclamide suppresses microglial M1 polarization, neurodegeneration, synaptic loss and α-synuclein phosphorylation as well as spatial learning deficits in P + M-injected mice. **A** Representative blots of NLRP3, active caspase-1 and mature IL-1β in the hippocampus. **B** Quantification of the blots. **C** Representative pictures of Iba-1 staining in the hippocampus and quantification of Iba-1 optical density. *n* = 3. **D**, **E** mRNA levels of M1 (**D**) and M2 (**E**) genes. *n* = 6. **F** Quantification of Neu-N + cells. **G** Quantification of PSD-95 optical density. **H** Representative blots and quantification of Neu-N, PSD-95, TH, total and Ser129-phosphorylated α-synuclein in mice. *n* = 6. **I** Escape latency, **J** traveled distance and (**K**) swimming speed of mice. *n* = 15–16; *p < 0.05, **p < 0.01; Scale bar = 50 μm

Subsequently, glybenclamide, a NLRP3 inflammasome inhibitor, was administered to mice intoxicated with P + M. Glybenclamide attenuated expressions of NLRP3, active caspase-1 and IL-1β in P + M-injected mice (Fig. 7A, B), indicating mitigated NLRP3 inflammasome activation. Microglial activation elicited by P + M was also suppressed by glybenclamide by showing reduced Iba-1 immunostaining density and ramified morphology of microglia in glybenclamide and P + M co-injected mice in comparison with P + M alone-treated mice (Fig. 7C).

Activated microglia can be divided into M1 (classic) phenotype to release a myriad of proinflammatory cytokines and M2 (alternative) phenotype to resolve inflammation, respectively [49]. Glybenclamide treatment abrogated P + M-elevated gene transcripts of two M1 microglial markers, iNOS and TNFα in mice (Fig. 7D). While, glybenclamide failed to confront P + M-elicited reduction of Arg-1 and YM-1 (M2 marker) genes in mice, indicating that glybenclamide blocks microglial M1 but not M2 polarization (Fig. 7E).

### Glybenclamide blocks neuronal damage and ameliorates cognitive dysfunction elicited by P + M

Glybenclamide treatment reduced P + M-elicited neuronal damage by showing recovered Neu-N^+^ neuron and PSD-95 expression in mice (Fig. 7F, G; Additional file 1: Fig. S4). Consistently, elevated expressions of Neu-N, PSD-95 and TH were also detected in glybenclamide and P + M co-injected mice in comparison with P + M alone group in Western blot (Fig. 7H). Furthermore, glybenclamide treatment reduced P + M-elicited expression of total and phosphorylated (Ser129) α-synuclein in mice (Fig. 7H).

We next investigated whether glybenclamide could ameliorate learning dysfunction in P + M-injected mice. In comparison with P + M alone, glybenclamide and P + M cotreatment reduced escape latency and traveled distance in mice, indicating improved learning capacity (Fig. 7I, J). We also determined the swimming speed of mice and revealed no difference among groups (Fig. 7K).

## Discussion

Our study supported that Mac1 is involved in cognitive impairment in a mouse PD model generated by P + M injection. The following salient features are reported: (1) the expression level of Mac1 was increased in P + M-injected mice and Mac1 knockout significantly alleviated P + M-elicited learning and memory impairments in mice; (2) Mac1 knockout attenuated neuronal damage, synapse loss and phosphorylation (Ser129) of α-synuclein elicited by P + M; (3) Mac1 mediates P + M-provoked microglial NLRP3 inflammasome activation; (4) NOX, especially NOX1 and NOX2, and downstream MAPK and PAK1 signals contributed to Mac1-mediated NLRP3 inflammasome activation; (5) pharmacological inactivation of NLRP3 inflammasome mitigated P + M-elicited microglial M1 activation and neuronal damage, which were associated with improved cognitive function of mice.

Traditionally, PD is believed as a movement disease characterized by nigral dopaminergic neurodegeneration [21, 50]. In contrast, recent evidence indicated that the pathology of PD extends to multisystem and patients exhibit not only motor abnormality but also a variety of NMS, including learning and memory impairments [9, 51]. Postmortem studies showed that limbic and cortical damage is correlated with poor cognition in PD [43, 52]. The mechanism underlying cognitive dysfunction in PD remains to be elucidated; however, abnormal activated microglia and related neuroinflammatory response have been considered to be involved in cognitive impairments in neurodegenerative process. Dys-regulated activation of microglia has been shown to exacerbate protein aggregation and synapse loss and thereafter impairing neuroplasticity in brain regions that related to cognition function [53–55]. Recently, we revealed microglial activation, hippocampal neurodegeneration and cognitive decline in P + M-generated mouse model of PD [9]. Here, we recognized Mac1 as an important receptor to mediate microglial activation and subsequent learning and memory impairments in the two pesticide-injected mice. We found that genetic deletion of Mac1 significantly mitigated P + M-provoked microglial activation, which was associated with mitigated neurodegeneration, synapse loss and phosphorylation (Ser129) of α-synuclein. More importantly, Mac1 deficiency ameliorated learning and memory impairments elicited by P + M, suggesting a critical role of Mac1-mediated microglial activation in cognitive deficits in PD. Consistently, Hong et al. found that mice with Mac1 deficiency were more resistant to synapse loss and neuronal damage induced by β-amyloid than WT mice [56]. In Zhang’s study, Mac1 deficiency also reduced hypoxia and LPS-induced damage of synaptic plasticity that related to learning and memory [57].

We also addressed the question regarding how Mac1 regulates microglial activation. NLRP3 inflammasome expresses high levels in activated microglia [58]. NLRP3 inflammasome activation promptly provokes caspase-1 activation and subsequent secretion of cytokines, IL-1β and IL-18 [58]. The importance of NLRP3 inflammasome in regulating microglial activation and related neurodgeneration has been revealed in several neurodegenerative disorders. In APP/PS1 double transgenic mouse model, NLRP3 inflammasome is activated in the hippocampus and knockout of NLRP3 or caspase-1 genes abrogates microglial M1 activation, neurodegeneration and learning and memory deficits [45]. Induced expression of an active form of NLRP3 in microglia exacerbates neurodegeneration of dopaminergic neurons and motor abnormality in MPTP-injected mice [59]. Moreover, α-synuclein has been found to damage dopaminergic neuron by stimulating activation of microglial NLRP3 inflammasome [60, 61]. In this study, P + M elicited NLRP3 inflammasome activation in mice. Genetic ablation or pharmacological inactivation of Mac1 significantly prevented P + M-elicited microglial NLRP3 inflammasome activation. Moreover, inactivation of NLRP3 inflammasome by glybenclamide confronted P + M-elicited microglial M1 activation. Importantly, glybenclamide alleviated hippocampal neurodegeneration, synaptic damage, phosphorylation (Ser129) of α-synuclein, and learning and memory dysfunction elicited by P + M, suggesting that NLRP3 inflammasome bridges Mac1 and downstream microglial activation and cognitive deficits.

NOX, a complex enzyme in microglia responsible for superoxide production, is able to regulate NLRP3 inflammasome activation. It has been found that β-amyloid could stimulate activation of NLRP3 inflammasome through NOX-derived superoxide in LPS-pretreated ARPE-19 cells [62]. In bone-marrow-derived macrophage, genetic ablation of NOX2 cytosolic subunit p47^phox^ attenuates NLRP3 inflammasome activation stimulated by multi-walled carbon nanotubes [63]. Consistently, in this study, inhibition of NOX, especially NOX1 and NOX2, attenuated NLRP3 inflammasome activation induced by P + M. More importantly, stimulating activation of NOX by PMA recovered NLRP3 inflammasome activation in Mac1 blocking peptide RGD and P + M co-treated microglia, suggesting that NOX is a key for the regulatory effects of Mac1 on NLRP3 inflammasome activation. Although how NOX mediates Mac1-regulated NLRP3 inflammasome activation remains unclear, NOX is known to be able to induce activation of MAPK signaling pathway through elevation of iROS [32]. MAPK, including ERK, JNK and p38 members, can phosphorylate NLRP3 and then affect NLRP3 inflammasome activation [64–66]. Here, inactivation of NOX by apocynin significantly reduced activation of MAPK in P + M-treated BV2 microglia. In addition, our results also showed that apocynin abrogated P + M-induced activation of PAK1, a kinase that can control NLRP3 inflammasome activation through phosphorylation of NLRP3 and pro-caspase-1 [48, 67]. Together, our findings suggested that MAPK and PAK1 are two critical downstream signals of NOX to regulate NLRP3 inflammasome activation.

Notably, it is well-documented that in addition to Mac1, other signaling molecules, such as RAGE and TLR, are also involved in microglial activation in response to environmental toxins, including paraquat [68–70]. Actually, the interactions among Mac1, TLR and RAGE have been reported in previous reports. Experimental evidence revealed an important role of Mac1 in TLR or RAGE-mediated inflammation [71–73]. However, the role and compensatory effects of TLR, RAGE and their interactions with Mac1 in microglia-mediated neuroinflammation in response to P + M exposure are still worth to be investigated to clarify the molecular mechanisms of P + M-induced neuroinflammation. Further study focusing on this point should be guaranteed. In addition, in the current study, all the experiments were performed in P + M-induced mouse PD model. As we know the fact that PD is a very complex disease and rodent PD models have limitations to completely mimic human PD features. For example, P + M-induced mouse PD model has received criticism due to its limited degree of cell death, variable loss of striatal dopamine content and different exposure routine with human [74, 75]. Therefore, whether the results and conclusion generated from this model could be extrapolated to humans need to be verified further using other rodent PD models and even human samples in the future.

## Conclusion

Altogether, this study recognized Mac1 as a key in cognitive deficits through NOX–NLRP3 inflammasome axis-dependent neuroinflammation in a mouse PD model. This adds to the understanding of cognitive dysfunction in PD and may provide a novel therapeutic strategy for combating this disorder.

## Supplementary Information

**Additional** **file** **1:** **Fig.** **S1.** P + M exposure elevates Mac1 expression in cortex of mice. The representative images of Mac1 staining in the cortex of mice at the indicated timepoints of P + M injection and quantification of Mac1 immunostaining density. *n* = 3; **p < 0.01; Scale bar = 100 μm. **Fig. S2**. Mac1 knockout attenuates P + M-induced neurodegeneration in the cortex of mice. (A) Quantification of Neu-N^+^ cell number in the cortex of mice. (B) Quantification of PSD-95 immunostaining in the cortex of mice. *n* = 4; **p < 0.01; Scale bar = 200 μm. **Fig.** **S3**. NOX contributes to Mac1-mediated NLRP3 inflammasome activation in primary microglia intoxicated with P + M. (A) Representative blots of NLRP3, active caspase-1 and mature IL-1β in P + M-treated primary cells with or without anti-Mac1 blocking Ab and the quantification of density of these blots. (B) Representative blots of NLRP3, active caspase-1 and mature IL-1β in P + M-treated primary cells with or without apocynin and the quantification of density of these blots. (C) Representative blots of NLRP3, active caspase-1 and mature IL-1β in P + M-treated primary cells with or without combined anti-Mac1 blocking antibody and PMA and the quantification of density of these blots. (D) Representative blots of phosphorylated and nonphosphorylated ERK1/2 in P + M-treated primary cells with or without apocynin and the quantification of density of these blots. *n* = 3; *p < 0.05, **p < 0.01. **Fig. S4**. Glybenclamide attenuates P + M-induced neurodegeneration in cortex of mice. (A) Quantification of Neu-N^+^ cell number in the cortex of mice. (B) Quantification of PSD-95 immunostaining in the cortex of mice. *n* = 4; **p < 0.01; Scale bar = 200 μm.

## Data Availability

All data generated or analyzed during this study are included in this published article [and its Additional files].

## Abbreviations

DAB: 3,3′-Diaminobenzidine
Iba-1: Ionized calcium binding adaptor molecule-1
IL-1β: Interleukin-1β
Mac1: Macrophage antigen complex-1
MAPK: Mitogen-activated protein kinases
MWM: Morris water maze
PAK1: P–P21-activated kinase 1
PFA: Paraformaldehyde
PD: Parkinson’s disease
PMA: Phorbol myristate acetate
NMS: Non-motor symptom
NOX: NADPH oxidase
PSD-95: Postsynaptic density protein 95
TH: Tyrosine hydroxylase (TH)
TNFα: Tumor necrosis factor α
