# Supplementary material for: Integrin Mac1 mediates paraquat and maneb-induced learning and memory impairments in mice through NADPH oxidase–NLRP3 inflammasome axis-dependent microglial activation
Source: J Neuroinflammation. 2023 Feb 18;20:42. doi: 10.1186/s12974-023-02732-x (PMC9938991; doi:10.1186/s12974-023-02732-x)
Supplement: Supplementary file 1 — Additional file 1: Fig. S1. P + M exposure elevates Mac1 expression in cortex of mice. The representative images of Mac1 staining in the cortex of mice at the indicated timepoints of P + M injection and quantification of Mac1 immunostaining density. n = 3; **p < 0.01; Scale bar = 100 μm. Fig. S2. Mac1 knockout attenuates P + M-induced neurodegeneration in the cortex of mice. (A) Quantification of Neu-N+ cell number in the cortex of mice. (B) Quantification of PSD-95 immunostaining in the cortex of mice. n = 4; **p < 0.01; Scale bar = 200 μm. Fig. S3. NOX contributes to Mac1-mediated NLRP3 inflammasome activation in primary microglia intoxicated with P + M. (A) Representative blots of NLRP3, active caspase-1 and mature IL-1β in P + M-treated primary cells with or without anti-Mac1 blocking Ab and the quantification of density of these blots. (B) Representative blots of NLRP3, active caspase-1 and mature IL-1β in P + M-treated primary cells with or without apocynin and the quantification of density of these blots. (C) Representative blots of NLRP3, active caspase-1 and mature IL-1β in P + M-treated primary cells with or without combined anti-Mac1 blocking antibody and PMA and the quantification of density of these blots. (D) Representative blots of phosphorylated and nonphosphorylated ERK1/2 in P + M-treated primary cells with or without apocynin and the quantification of density of these blots. n = 3; *p < 0.05, **p < 0.01. Fig. S4. Glybenclamide attenuates P + M-induced neurodegeneration in cortex of mice. (A) Quantification of Neu-N+ cell number in the cortex of mice. (B) Quantification of PSD-95 immunostaining in the cortex of mice. n = 4; **p < 0.01; Scale bar = 200 μm. [file 12974_2023_2732_MOESM1_ESM.doc]

**Additional file 1**

**
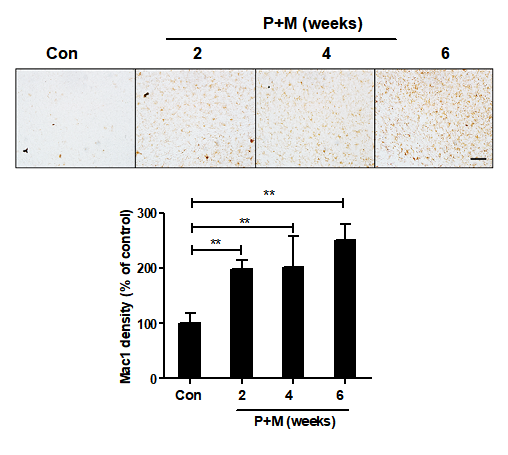
**

**Fig. S1. P+M exposure elevates Mac1 expression in cortex of mice.** The representative images of Mac1 staining in the cortex of mice at the indicated time points of P+M injection and quantification of Mac1 immunostaining density. n = 3; ***p*<0.01; Scale bar = 100 μm.


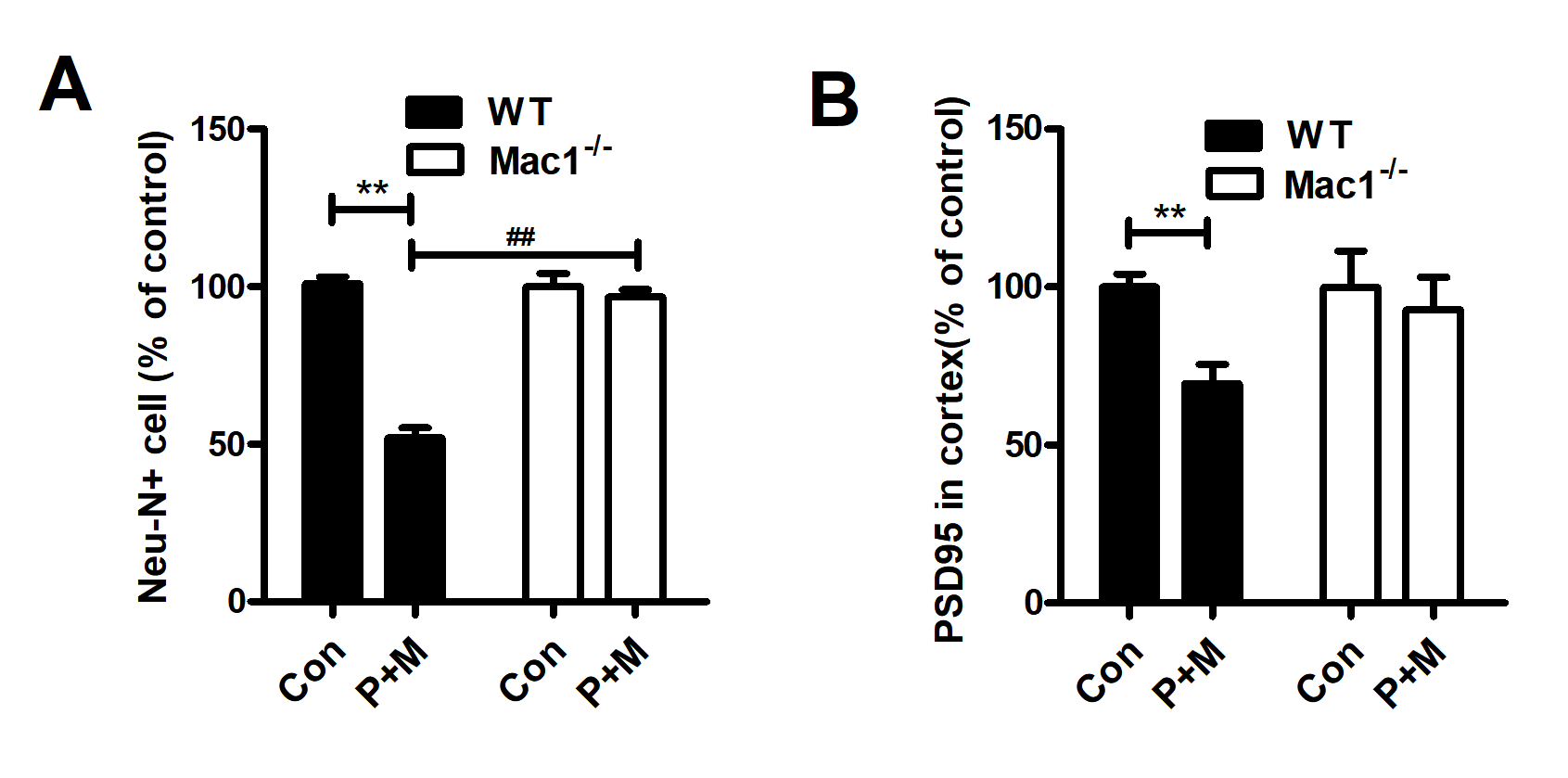


**Fig. S2. Mac1 knockout attenuates P+M-induced neurodegeneration in the cortex of mice.** (A) Quantification of Neu-N^+^ cell number in the cortex of mice. (B) Quantification of PSD-95 immunostaining in the cortex of mice. n = 4; ***p*<0.01.


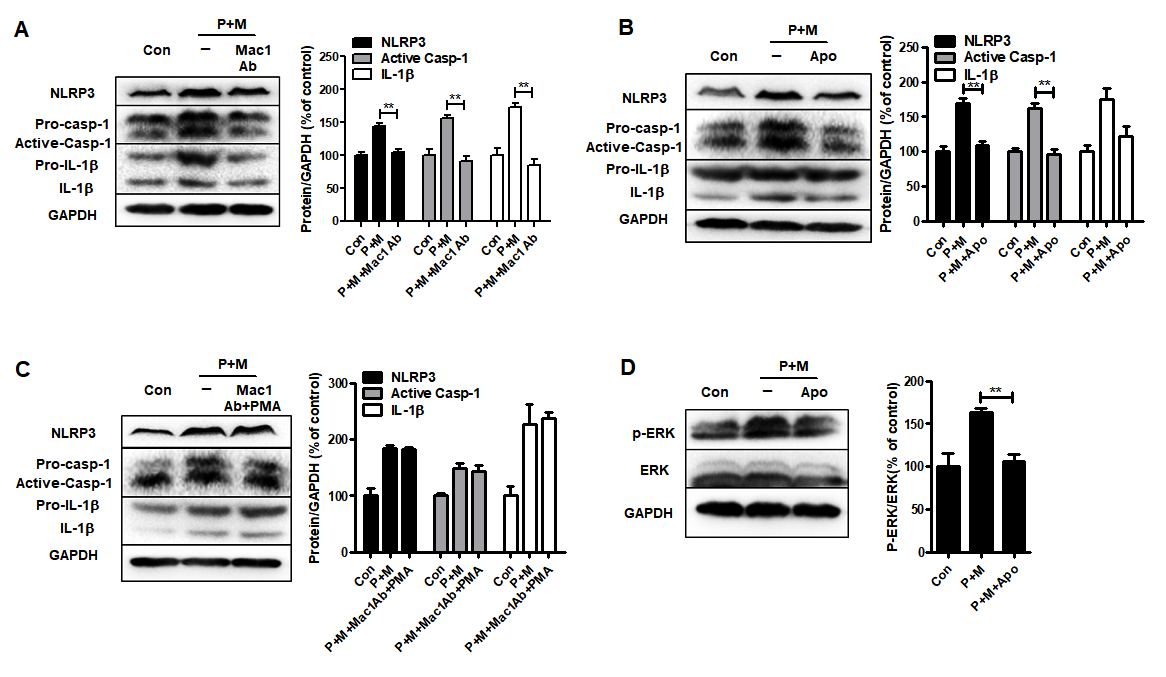


**Fig. S3. NOX contributes to Mac1-mediated NLRP3 inflammasome activation in primary microglia intoxicated with P+M.** (A) Representative blots of NLRP3, active caspase-1 and mature IL-1β in P+M-treated primary cells with or without anti-Mac1 blocking Ab and the quantification of density of these blots. (B) Representative blots of NLRP3, active caspase-1 and mature IL-1β in P+M-treated primary cells with or without apocynin and the quantification of density of these blots. (C) Representative blots of NLRP3, active caspase-1 and mature IL-1β in P+M-treated primary cells with or without combined anti-Mac1 blocking antibody and PMA and the quantification of density of these blots. (D) Representative blots of phosphorylated and nonphosphorylated ERK1/2 in P+M-treated primary cells with or without apocynin and the quantification of density of these blots. n = 3; *p< 0.05, **p< 0.01.


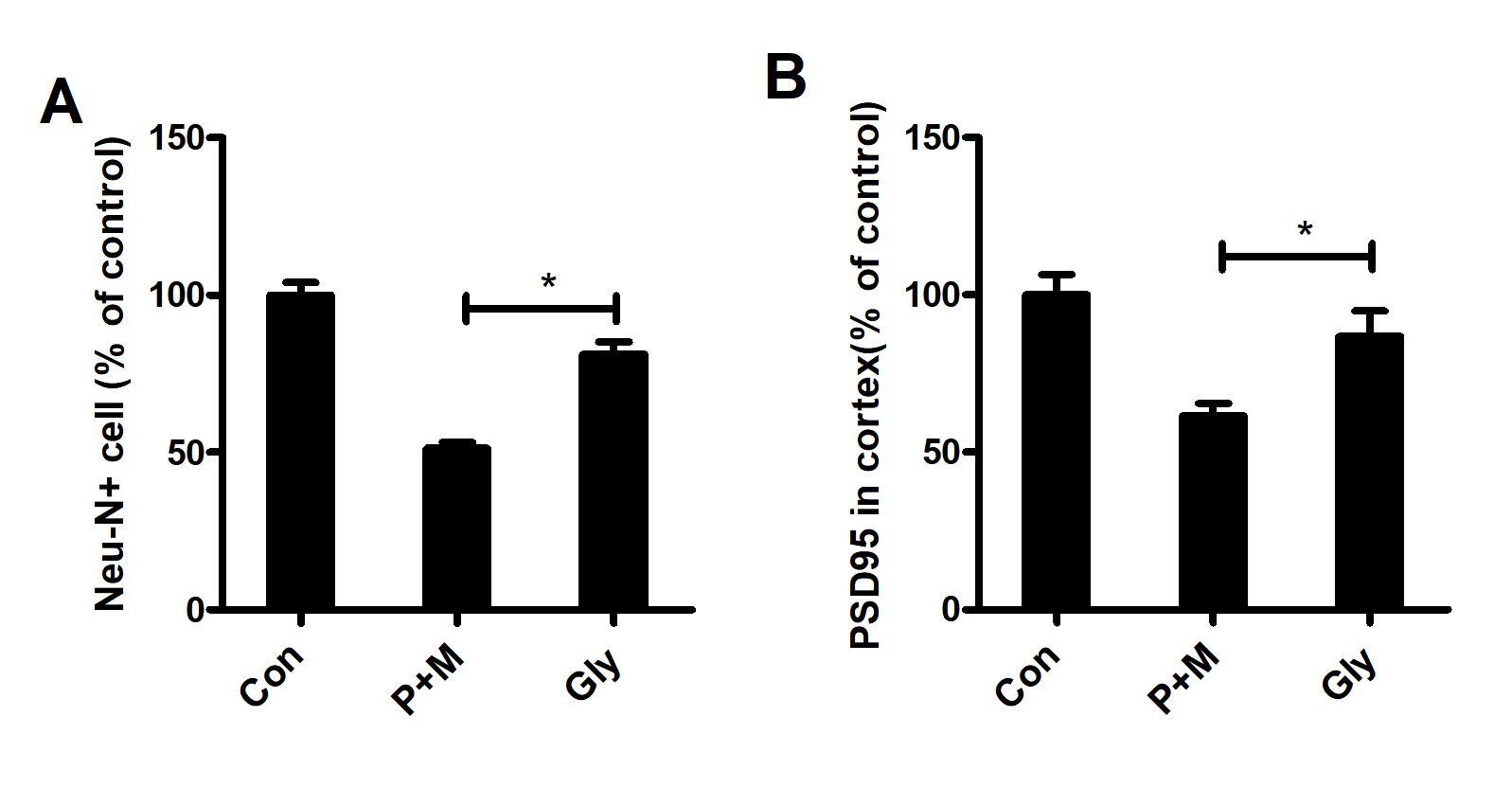


**Fig. S4. Glybenclamide attenuates P+M-induced neurodegeneration in cortex of mice.** (A) Quantification of Neu-N^+^ cell number in the cortex of mice. (B) Quantification of PSD-95 immunostaining in the cortex of mice. n = 4; ***p*<0.01.
